# Femoral supracondylar focal dome osteotomy with plate fixation for acute correction of frontal plane knee deformity

**DOI:** 10.1007/s11751-015-0218-2

**Published:** 2015-04-10

**Authors:** Sherif Ahmed El Ghazaly, El-Hussein Mohamed El-Moatasem

**Affiliations:** Orthopedic Department, Al-Demerdash Hospital, Ain Shams University, Abbassia Square, Abbassia, 11381 Cairo, Egypt

**Keywords:** Knee valgus, Varus knee, Frontal knee deformity, Focal osteotomy, Femoral osteotomy, Dome osteotomy

## Abstract

Focal dome osteotomy (FDO) allows deformity correction without secondary translational deformity. The purpose of this study was to evaluate the degree of correction and knee functional outcome after correction of frontal knee deformity using femoral supracondylar FDO fixed with plate and screws. A prospective study included 12 consecutive cases of femoral frontal plane deformity that underwent correction using supracondylar focal osteotomy fixed by plate and screws. Average age was 27 years, while mean follow-up was 2.1 years. Functional assessment was done using the Hospital for Special Surgery (HSS) knee score. The HSS knee score improved from 85 to 96.8 points. Desired correction was achieved in all cases. Postoperative mechanical axis analysis on long film and scanogram showed no secondary deformity. The overall postoperative mechanical axis was at 3.2 mm medially (range 2–5 mm). Autogenous bone graft was not used in any case, and uneventful osteotomy union was achieved at a mean of 13.8 weeks. Minor complications were encountered in two cases. There were no implant failures or reoperations. Supracondylar FDO of the femur with plate fixation is a reproducible technique that can produce full correction of distal femoral frontal plane deformity, while avoiding creating a secondary deformity. Knee function was improved with good patient satisfaction.

## Introduction

Frontal plane knee deformity may be idiopathic or secondary to trauma or osteomalacia. Tibio-femoral premature arthritis can occur from single compartment loading with patellofemoral arthritis in valgus knees. An angular correction by wedge corrective osteotomy is the standard treatment. Some drawbacks of the wedge osteotomy are limb length discrepancy, a mismatch of fragment ends created by osteotomy and the need for translation of the distal fragment. Circular bone cuts allow deformity correction without introducing a length discrepancy, mismatch or need for segment translation. A dome osteotomy (DO) is a cylindrical osteotomy [1, 2], with the corresponding bone cuts rotating around the central axis of the cylinder [3]. No bone is resected, and this avoids a length discrepancy. Brackett [4] described a dome osteotomy of the proximal femur to treat an ununited fracture of the femoral neck. A focal dome osteotomy (FDO) allows deformity correction while avoiding the need to produce a translation at the osteotomy in order to realign the proximal and distal axes [2, 3, 5]. We hypothesized that a FDO and plate fixation may be used in the distal femur to ensure full deformity correction without a secondary deformity and with maximal contact at the osteotomy, which allows improved healing and function.

## Materials and methods

**Fig. 1:**
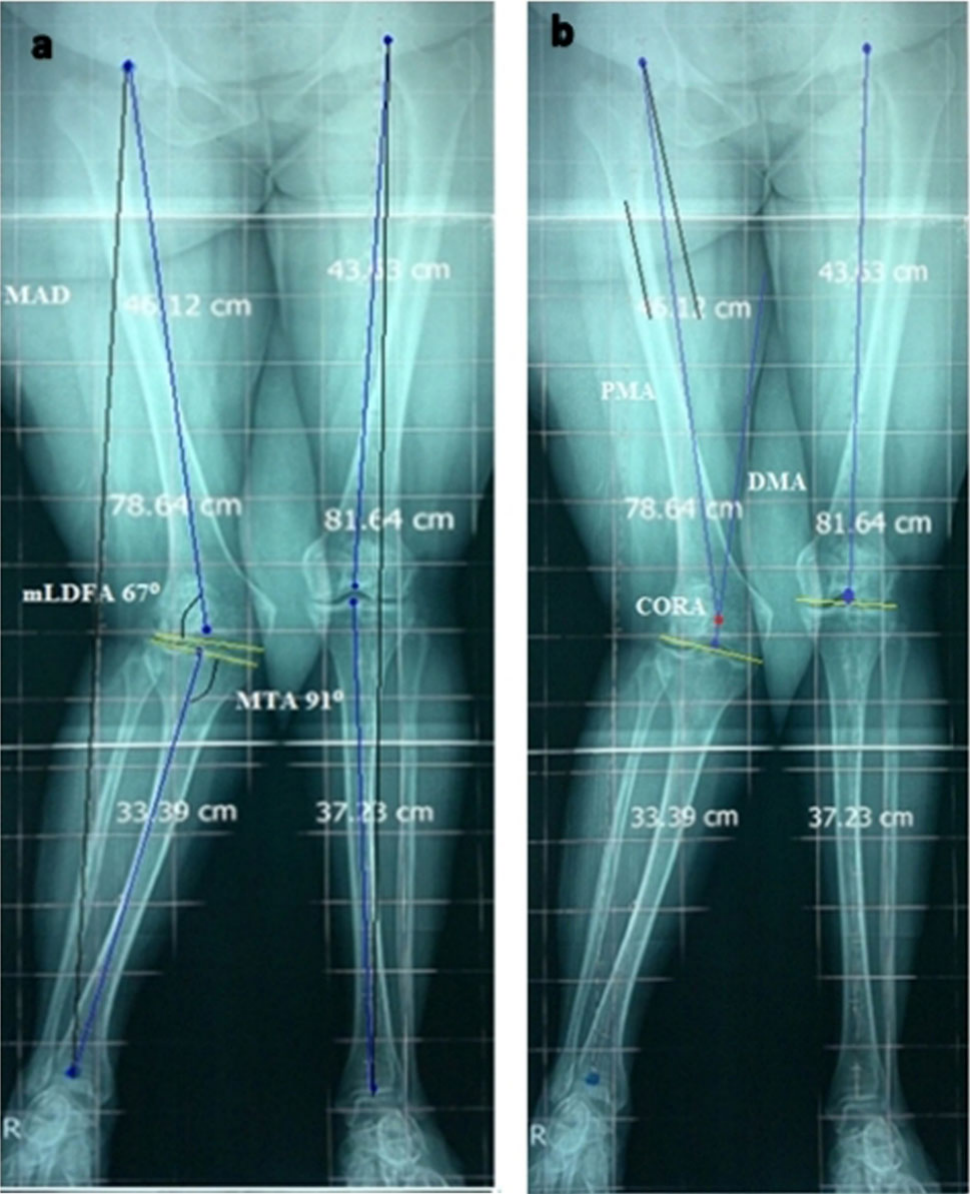
**a** Preoperative planning (case 2) showing lateral mechanical axis deviation with normal mMPTA 91° and mLDFA 67° so the patient had genu valgum of femoral origin; **b** CORA was determined by intersection of the proximal mechanical axis (PMA) (a line 7° valgus to the anatomical axis, extending from the centre of the hip) and the distal mechanical axis (DMA), which was measured at a mLDFA of 88° (the contralateral knee was taken as the normal)

From July 2010 until December 2012, a prospective observational study was conducted of 12 cases on frontal knee deformity treated using a supracondylar FDO. Inclusion criteria were frontal plane deformity causing mechanical axis deviation, mechanical lateral distal femoral angle (mLDFA) below 84° or above 95° (Fig. 1), normal medial proximal tibial angle (MPTA) and anterior knee pain (retropatellar and/or joint line). Cases with combined femoral and tibial deformity were excluded.

There were seven males and five females with average age 27 years (range 16–38). The study was reviewed and approved by the university ethical committee. All patients gave their informed consent. There were nine valgus knees and three varus knee deformities with five knees showing radiological evidence of arthritis. Causes of deformity were traumatic (four), idiopathic (six) and osteomalacia (two cases).

### Preoperative planning

All patients had antero-posterior long-limb films and a CT scanogram. Mechanical femoro-tibial angle (mFTA) was measured on long-limb films, while scanograms were used to confirm mechanical axis deviation (MAD) using the mal-alignment test and to define the magnitude of deviation. MAD was measured in millimetres from the knee centre. For every patient, the magnitude of femoral deformity was defined via the mLDFA, while the centre of rotation of angulation (CORA) was radiologically defined (Fig. 1). The preoperative MAD ranged from 86 mm lateral to 20 mm medial. The average preoperative mFTA was 18.5° in cases of valgus deformity and 18° in varus cases. The average angular deformity was 11.5° (range 8–19) and mLDFA 75.2° (range 67°–82°) in valgus cases; 25° (range 24°–26°) and mLDFA 102° (range 100°–104°) in varus cases (Table 1).

**Table 1:** Demographic, preoperative and postoperative alignment data for all patients

No.	Varus/valgus	Age	Sex	m F-T angle preop	m F-T angle postop	Deformity angle	Angle corrected	Preop mLDFA	Postop mLDFA	Preop MAD	Postop MAD
1	Val	16	M	15	5	8	10	82	93	+48 mm	−3 mm
2	Val	30	F	26	6	19	20	67	87	+86 mm	−2 mm
3	Val	32	M	18	7	11	11	76	91	+54 mm	−5 mm
4	Var	38	M	18	6	25	24	102	94	−20 mm	−3 mm
5	Val	17	F	15	5	8	10	79	93	+49 mm	−3 mm
6	Val	32	F	18	5	11	13	75	94	+56 mm	−4 mm
7	Val	35	M	19	7	12	12	77	91	+54 mm	−3 mm
8	Var	28	M	17	5	24	22	100	93	−19 mm	−3 mm
9	Val	34	M	21	6	14	15	72	90	+56 mm	−4 mm
10	Var	24	F	19	4	26	23	104	93	−20 mm	−3 mm
11	Val	22	M	17	5	10	12	75	93	+55 mm	−3 mm
12	Val	21	F	18	6	11	12	74	88	+53 mm	−2 mm
Mean	–	27 (16–38)	M:7	Val:18.5	Val:5.75	Val: 11.5	Val: 12.77	Val:75.2	Val:91	Val:56.75	Val:3.25
			F:5	Var:18	Var:5	Var:25	Var:23	Var:102	Var:94	Var:20	Var:3

### Operative procedure

**Fig. 2:**
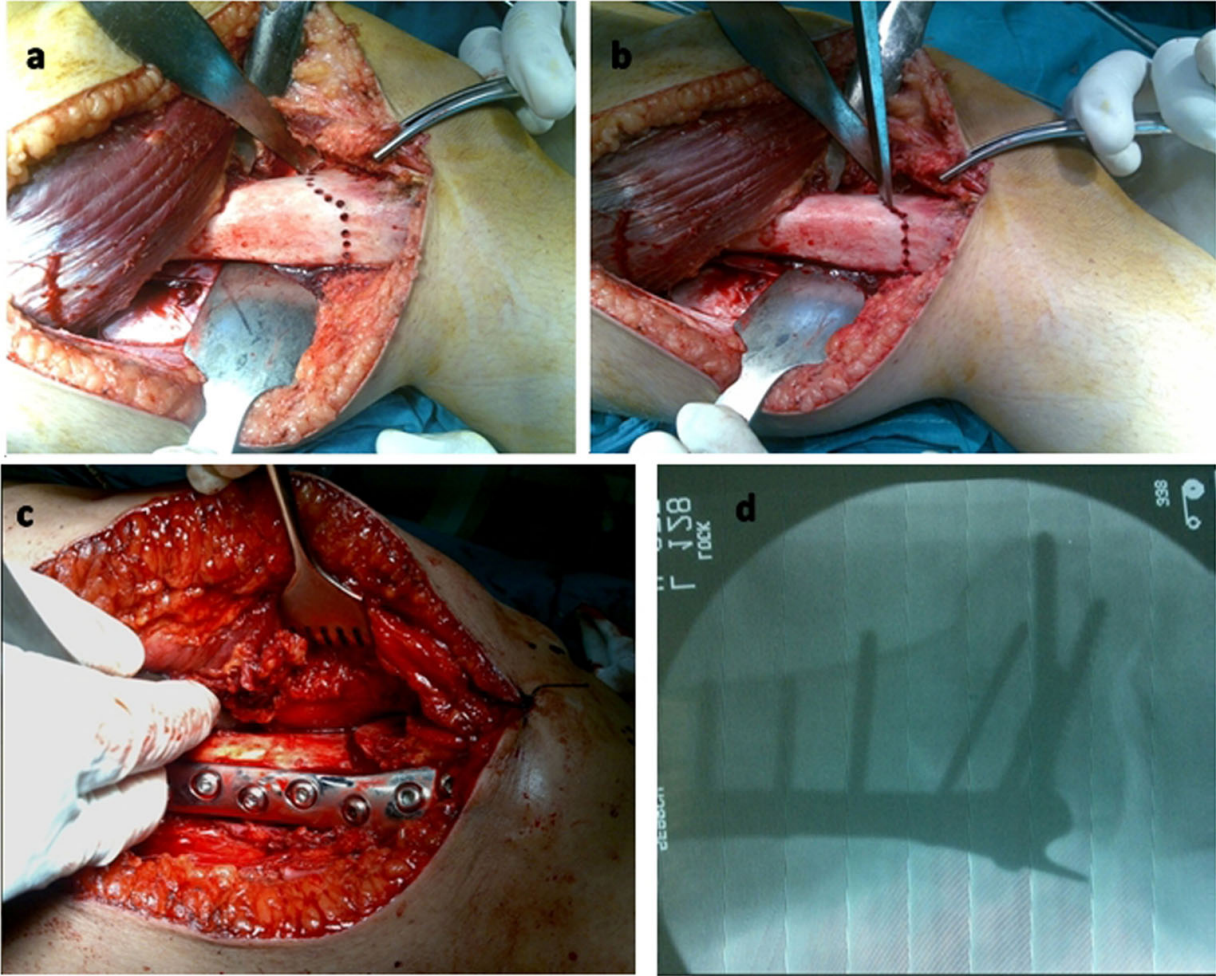
**a** Intraoperative photo showing exposure of the supracondylar region of the femur and drilling of the planned dome osteotomy; **b** intraoperative photo showing connecting the predrilled holes using the osteotome; **c** intraoperative photo showing correction of deformity and plate and screw fixation; **d** intraoperative fluoroscopy showing correction of deformity, minimal translation and plating

Patients received general anaesthesia and were operated in the supine position. A second generation cephalosporin was given at induction. A pneumatic tourniquet was applied and inflated to 350 mmHg. The C-arm was positioned in order to obtain a clear view of the distal half of the femur. Two markers were placed at the mid-inguinal point corresponding to the femoral pulse and at the mid-ankle position. Using a 15- to 20-cm lateral incision and posterolateral approach, the vastus lateralis was elevated from the lateral intermuscular septum. The lateral and anterior femoral cortices in the supracondylar area were exposed. A blunt-tipped Hohmann retractor was placed medially exposing the anteromedial cortex, and a second wide retractor was placed subperiosteally posterior to the distal femur to protect the vascular bundle (Fig. 2). At the selected site (metaphyseal area, 1 finger breadth above the medial femoral condyle), a circular line was drawn using electrocautery. A 2.5-mm drill bit and drill sleeve were used to create drill holes along the circular line, just penetrating the posterior cortex. The holes were created close to each other to ensure a smooth circular shape. The osteotomy was convex superior, creating a FDO (Fig. 2a). A ¼ in. osteotome was then used to connect the drill holes, except at the corners, where a curved bone gauge was used (Fig. 2b). Before completing the osteotomy, the plate to be used for fixation (7–8 holes femoral buttress condylar or humeral “T” plate) was contoured to the desired degree of correction.

**Fig. 3:**
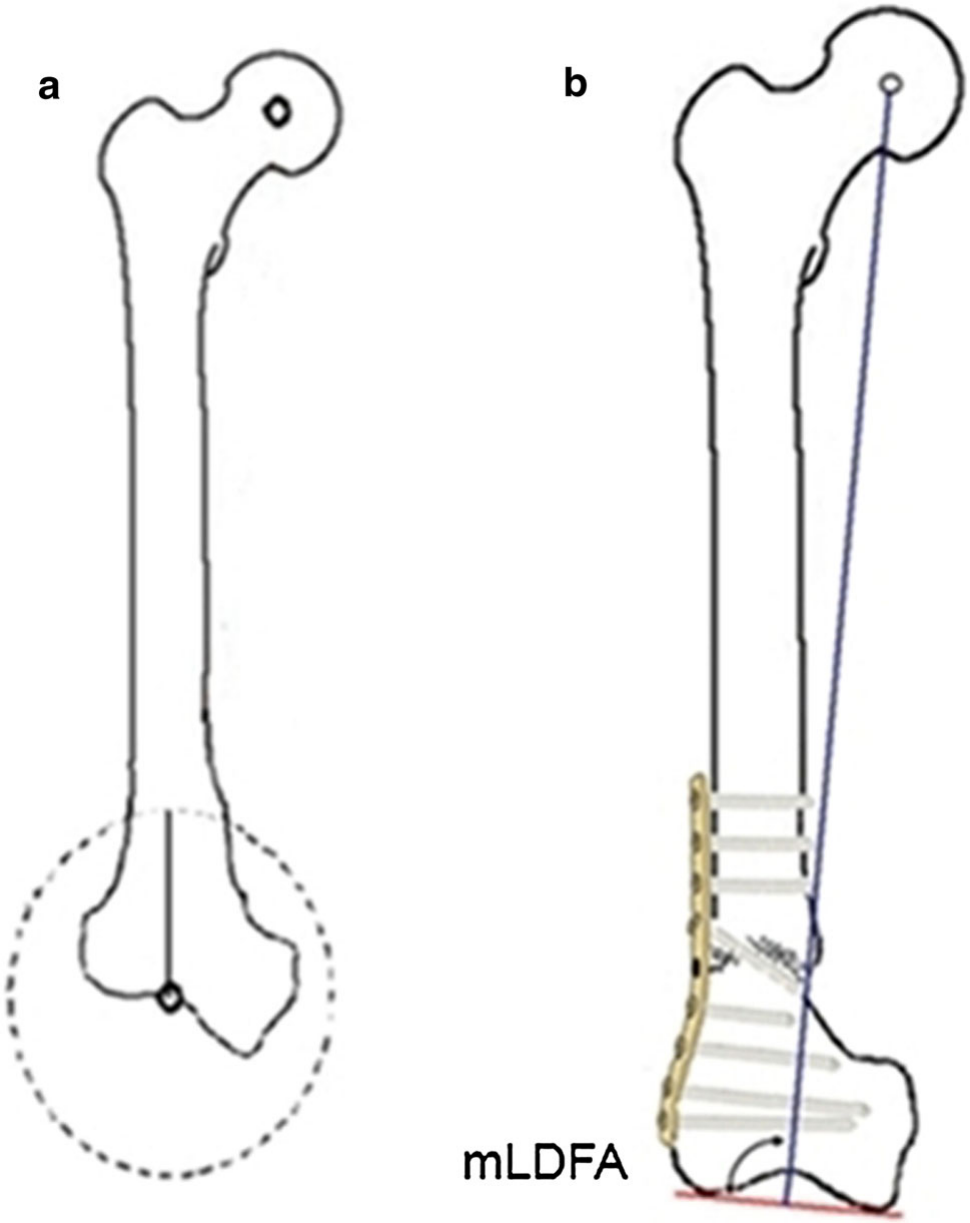
**a** Schematic drawing showing the CORA-based planning of focal dome osteotomy; **b** drawing showing correction of deformity using focal dome osteotomy, minimal translation and plating with one screw transfixing the osteotomy

The plate was placed on the lateral cortex, and two drill holes immediately proximal to the osteotomy site were drilled and tapped. Carefully under C-arm control, a ½ in. osteotome was used to finalize the osteotomy medially, taking care not to advance the osteotome too far medial or too far posterior. With the plate in position and fixed with two screws, the osteotomy was completed slowly by osteoclasis. The leg was manipulated into varus or valgus according to initial deformity, until the knee joint line was horizontal and the deformity clinically corrected. The distal femoral fragment rotated along the osteotomy and was aligned under the plate. Deformity correction and overall limb alignment were checked under C-arm; an electrocautery cord was placed from the femoral head marker to the mid-ankle marker, and the knee was viewed to ensure that this line was brought in the knee centre or just lateral to the medial tibial spine. According to plate type, plate fixation was completed using 2–3 fully threaded cancellous screws (Figs. 2d, 3).

All patients followed the same rehabilitation protocol: gradual progress of return of knee range of motion and function by knee flexion and extension and quadriceps exercises beginning from third postoperative day. Ambulation was allowed using two axillary crutches, but no weight-bearing was permitted for the first 3 weeks. This was followed by toe-touch walking for 2 weeks, and at 5 weeks, one axillary crutch was used for the next month with gradual transition to full weight-bearing.

## Results

The average follow-up was 2.1 years (range 1.6–2.5) years with regular monthly follow-ups. The patients were assessed using the Hospital for Special Surgery (HSS) knee score preoperatively, at 6 months and at final follow-up. Data were collected regularly into a special data collection sheet, entered into a computer for analysis and presented in the form of mean and ranges.

**Fig. 4:**
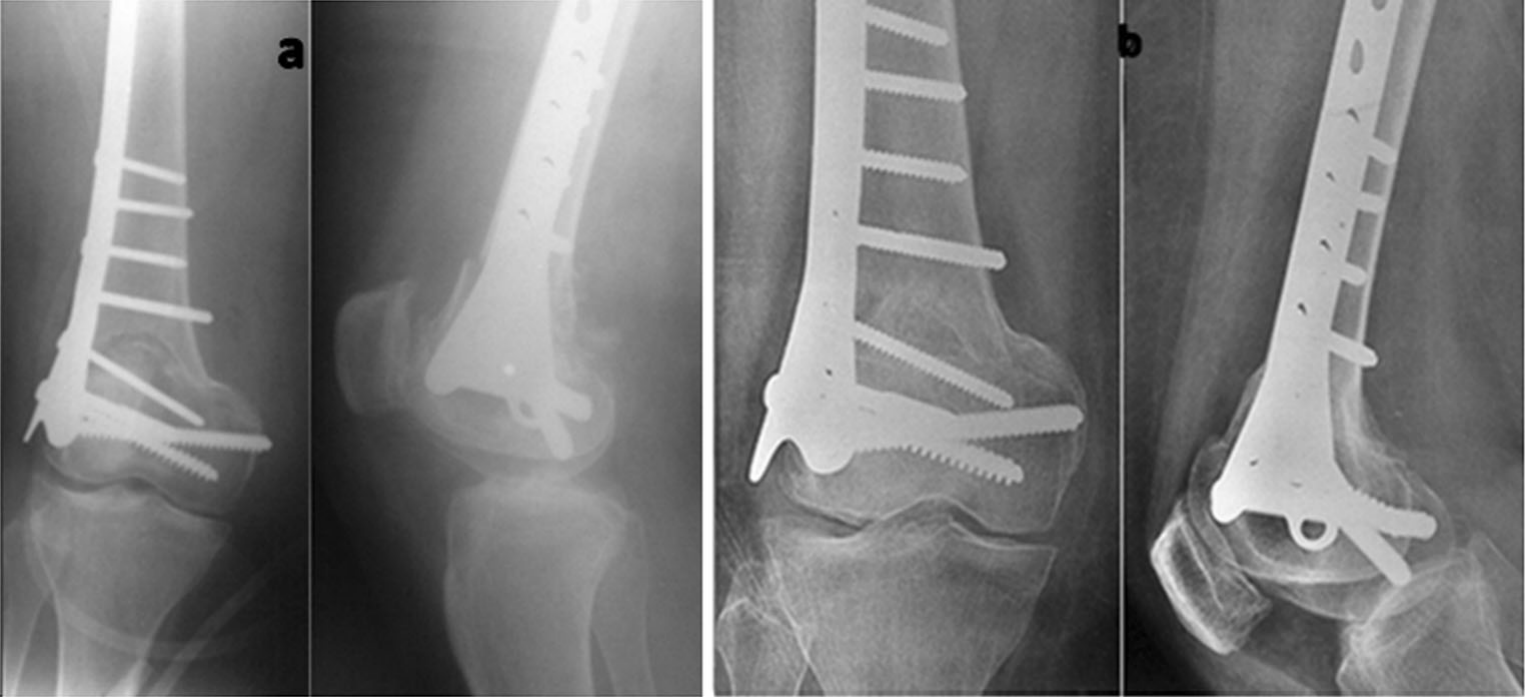
**a** X-ray of the distal femur showing full correction of deformity and plating. This patient had osteoarthritis; **b** X-ray 6 months postoperative showing full union

**Fig. 5:**
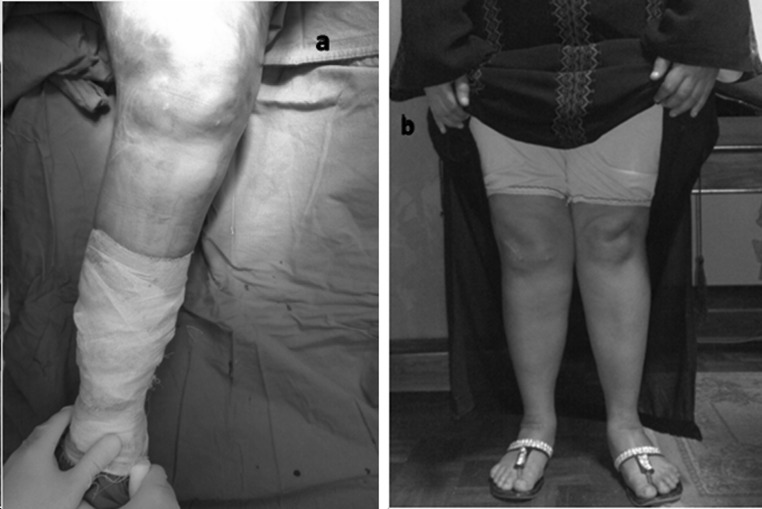
**a** Intraoperative photo showing severe genu valgum before correction; **b** clinical photo of the same case, 19 months later, showing full correction

All osteotomies united uneventfully (Fig. 4). The mean time to union was 13.8 weeks (range 12–16). The preoperative limb malalignment was corrected, and the desired correction was achieved in all cases (Fig. 5). The average angular correction for valgus deformities was 12.77° (range 10°–20°) and 23° (range 22°–24°) for varus deformities. In valgus deformities, the mechanical axis improved from an average preoperative value of 56.75 mm laterally to an average of 3.25 mm medially; the improvement for varus deformities was from 20 mm medially to 3 mm. The overall postoperative mechanical axis was at 3.2 mm medial to the centre of the knee (range 2–5 mm). Postoperatively, the mean mLDFA was 91° in valgus cases and 94° in varus cases (Table 1).

The mean preoperative HSS score was 85, and this improved to 96.8 at final follow-up. Patients were satisfied with the procedure. Two complications are reported: a case of superficial wound infection which improved on oral antibiotics and one patient complaining of local irritation of the iliotibial band over the end of a buttress condylar plate. This patient used warm packs and anti-inflammatory medication until plate removal. There were no cases of implant failure, and no cases required secondary intervention for union of the osteotomy.

## Discussion

Realignment osteotomy aims to correct limb alignment and may influence knee osteoarthritis [6]. Assessment of overall limb alignment was with the mechanical axis, while realignment of the knee was judged through correction of the joint orientation line. Deformity correction in the coronal plane may be achieved using wedge osteotomy or the DO [7]. According to Paley and Tetsworth [8, 9], DO is a cylindrical osteotomy with corresponding bone cuts, which rotate around the central axis of a circle. When this central axis corresponds with the CORA, this is called a FDO and correction of the deformity can be attained without translation of the bone axes [8, 9]. In our series, the preoperative radiographic planning showed the CORA to be at the knee joint or femoral condyle level in all cases. Using this CORA as the centre of a circle, the planned arc upon which the DO was created was based on the centre of this circle ensuring a FDO. Full correction was attained with minimal translation of the distal bone fragment (Fig. 3). Only cases with isolated femoral deformity were included for this observational series.

The dome osteotomy was chosen instead of a wedge osteotomy to avoid limb shortening in closing wedge corrections or a delayed union with more restrictive weight-bearing in opening wedge corrections (absent bone contact). The DO provides a large surface and maximizes bone contact, thereby ensuring optimal healing. Multiple drill holes followed by a low-energy osteotomy produces small bone spikes that interdigitate at the osteotomy after acute deformity correction; this can be compressed for added stability, reducing segment motion during osteotomy fixation and reducing the stress on the plate and screws. This caters for early partial weight-bearing, which was started at 3 weeks.

The DO allows high degrees of correction in the coronal plane. This was seen at preoperative planning and confirmed intraoperatively and on postoperative radiographs. Unlike Gugenheim and Brinker [6] who fixed the osteotomy using a retrograde femoral nail, we chose to use plates which are easily contoured. Deformity correction of up to 20° was accomplished with ease, and the osteotomy site remodelling resulted in an absence of secondary femoral deformity, which facilitates arthroplasty later.

Some authors describe an antero-medial approach, separating the vastus medialis and a medial knee arthrotomy [10]. We prefer the lateral approach as being more familiar and allows for an iliotibial band release, which is usually tight in valgus knees. The DO is technically demanding as performing the osteotomy as an arc needs care and precision to maintain the circular contour to ensure perfect segment rotation and bone contact and avoid inadvertent propagation. Gugenheim and Brinker [6] have described a percutaneous technique. We chose an open technique, which facilitates precision under direct vision and simpler plate fixation. Fixation of supracondylar osteotomies has been described using angled blade-plates, angle-stable plates or intramedullary nails [7, 11]. Wang and Hsu [12] chose to fix the osteotomy with a 90° angled blade-plate. We used a buttress condylar plate or “T” plate, avoiding the cost of more expensive or modern implants while retaining the ability to contour the plate (before osteotomy) to reach the desired correction. In adolescents, the antero-posterior dimensions of the distal femur are not large enough to place a condylar buttress plate, but we found the “T” plate suitable for fixation. Placing three screws in the distal segment and one transfixing, the osteotomy gave sufficient stability until osteotomy union. We found a transfixing screw important for all cases and provided the added stability as described by Wang and Hsu [12]. The “T” plate is a relatively weak implant in comparison with the condylar buttress plate, but we found it performed well for the selected cases and did not encounter implant failure.

Alternative techniques involve a lateral incomplete open-wedge osteotomy with use of spacer plates [7]. Some limitation of deformity correction is encountered by the limited ability to open a large wedge without need for bone grafts; this is in contrast to the greater versatility and rotational capacity of dome osteotomy.

We have used this technique to correct a coronal knee deformity in two patients near skeletal maturity with the average age 16.5 years unlike the work by Gugenheim and Brinker [6] who were not able to use their technique for skeletally immature patients. In one case (aged 16 years), the T plate was placed proximally such that no screws crossed the physis. In the other case (17 years), the plate was placed in the usual position and the screws distal to the osteotomy passed across the physis in effect fusing it.

In contrast to previous studies where the osteotomy was fixed using an external fixator [5], we have used plates and screws to avoid tethering of the quadriceps, pin track infections, loosening and the use of a cumbersome device. In a comparative study of internal versus external fixation for distal femoral osteotomies, Seah et al. [13] found no significant differences and concluded that the fixation method should be left to the discretion of the surgeon. This work coincides with the work of Wang and Hsu who used 90° blade-plates and screws for osteotomy fixation; the use of femoral condylar buttress or “T” plates is, in our view, easier and requires a less difficult after-care period.

Watanabe et al. [5] have stated that a DO with internal fixation cannot correct angulation precisely due to a difference in the centres of the deformity and that of the osteotomy. The current work has shown that, using the CORA method, executing a FDO for an epiphyseal CORA and realigning the mechanical axis to the knee centre can achieve deformity correction even when fixed using plate and screws.

Wang and Hsu [12] have reported an improvement in patellar tracking in patients with severe patellofemoral arthritis, which persisted to the time of the latest follow-up. Patellar tracking is improved because this corrective osteotomy effectively reduces the “Q” angle. To ensure proper patellar tracking after such osteotomy, it is important to avoid internal rotation of the distal femoral segment. The patients in this series noted an improvement in anterior knee pain.

The most common complications following corrective angular osteotomies are non-union and failure of fixation [12]. To achieve union, good bone apposition and stable fixation are required [12]. In our series, the use of plate and screws provided the required rigid fixation and allowed full union. We found no technique-related complications or peroneal neuropathy even after correction of high degrees of valgus deformity (>25°), in accordance with work by Gugenheim and Brinker [6], while Watanabe et al. [5] reported one case of transient peroneal neuropathy. Occasionally, mild under-correction may occur if careful planning and execution of surgical steps are not followed. This is in accordance with work by Gugenheim and Brinker [6], who reported no complications other than a single non-union and a single case of under-correction.

The limitations to this study are the small number of cases, and it is an observational study. There is no comparative cohort. Future studies involving a larger numbers of cases and those comparing the use of plates with an external fixator may be useful.

## Conclusion

This work shows that an acute angular correction of a distal femoral deformity by FDO, via a postero-lateral approach, can be used to correct either a varus or valgus knee deformity. This is achieved without muscle violation and has the added benefit of using a standard plate and screw fixation device, which is available in most hospitals worldwide. Rigid plate fixation and the absence of muscle division allow for an accelerated rehabilitation programme with early restoration of knee motion and function.
